# Concomitant Colonization of *Helicobacter pylori* in Dental Plaque and Gastric Biopsy

**DOI:** 10.1155/2014/871601

**Published:** 2014-07-09

**Authors:** Amin Talebi Bezmin Abadi, Ashraf Mohabati Mobarez, Omid Teymournejad, Mona Karbalaei

**Affiliations:** ^1^Department of Bacteriology, Faculty of Medical Sciences, Tarbiat Modares University, P.O. Box 14115-111, Tehran, Iran; ^2^Department of Microbiology, School of Medicine, University of Medical Science, Babol, Iran

## Abstract

Frequently reported *H. pylori* antimicrobial therapy failures suggest that there might be a different niche where the bacteria can stay safe. Current study aims to examine potential role of oral colonization of *H. pylori* to feed reinfection after primary therapy. However, patients who were admitted to the gastroscopy section were chosen and gastric biopsy and dental plaque specimens were collected. Molecular and biochemical tests were applied to confirm *H. pylori* identity in different colonization niches. Results showed that 88.8% of dyspeptic patients had epigastric pains with nocturnal awakening when they were hungry (*P* = 0.023). All patients who received therapy already were again H. pylori positive while they are still carrying *H. pylori* in dental plaque (*P* = 0.001). Moreover, H. pylori infection was sought in 100% of gastric biopsy's dyspeptic patients who had ulcerated esophagitis and erosive duodenitis and who were *H. pylori* positive, and 75% of dyspeptic patients with duodenum deformity had this bacterium in gastric biopsies (*P* = 0.004). Present study showed that only successful eradication of gastric *H. pylori* cannot guarantee prevention of reinfection. Conclusively, a new strategy which indicates concomitant eradication in oral and gastric colonization can result in clearance of *H. pylori* infection.

## 1. Introduction

*Helicobacter pylori* (*H. pylori*) is a Gram-negative, spiral, and motile bacterium that is present in the human stomach of approximately half of the world's population [1].* H. pylori* is an important gastrointestinal pathogen that is strongly associated with gastritis as well as peptic ulcer disease. There is strong evidence that* H. pylori* has an undeniable role in occurrence of gastric abnormality, atrophic inflammation, and gastric cancer [2]. Colonization begins in childhood; however, little is known about its timing and actual route of bacterial transmission [3, 4]. Recent findings are indicating a narrow link between oral and gastric colonization of* H. pylori* [5]. Frequently failed antibiotic therapy to cure* H. pylori* infection suggests that there might be certain different sites where the organism can survive [1, 6]. To date, the exact mechanism of transmission of* H. pylori* is not fully understood, a crucial fact which implies on unknown routes and reservoir locations that are still undiscovered. Indeed, defeated therapeutic approaches to cure gastric* H. pylori* infection triggered a thought that different locality might be involved in reinfection of this persistent bacterium [6]. Nonetheless, alarm symptoms and endoscopic finding are such approach to detect causes of dyspepsia [1, 7]. Dyspepsia is a kind of discomfort in center upper abdomen and can be affected by many factors such as peptic ulcer and gastroesophageal reflux [1]. Alarm symptoms have poor diagnosis for etiology in dyspeptic patients [8] and endoscopy is an invasive technique for diagnosis of gastritis, but that will not be enough for detecting the* H. pylori* [9]. As such, there are noninvasive tests such as urea breast test (UBT) and stool and blood tests which can be done in alignment with endoscopic method [10]. With regard to the diagnosis, the noninvasive tests are often used [10]. The aim of our study was to investigate the relationship between alarm symptoms and endoscopic findings with* H. pylori* colonization in dental plaque and gastric biopsy isolated from Iranian dyspeptic patients.

## 2. Materials and Methods

### 2.1. Questionnaire

In this survey, patients who admitted to the gastroscopy section at Baghiatallah hospital, Tehran, Iran, were chosen for our examination. Pain with or without nocturnal awakening while patient is hungry, bloating after meal with or without nocturnal awakening, reflux with or without nocturnal awakening, and endoscopic finding such as ulcerated or nonulcerated esophagitis, antral gastritis, erosive gastritis, gastric ulcer, duodenitis, duodenum ulcer, erosive duodenitis, and duodenum deformity were applied as including criteria.

### 2.2. Sampling

Gastric biopsy and dental plaque specimens were collected from each dyspeptic subject. Dental plaque and biopsy's sample were carried in thioglycolate broth (Merck, Germany) and then sent to the laboratory in less than four hours after gastric endoscopy. Two samples from antrum were shipped immediately after endoscopic procedures to microbiology and pathology labs for detecting* H. pylori* identity.

### 2.3. Bacterial Isolation

Dental plaques and gastric biopsies were cultured for one week in anaerobic jar with gas pack C (Merck, Germany) and presence of* H. pylori* was confirmed by biochemical tests: catalase, oxidase, urease, and gram staining. In brief, all samples were cultured in* Brucella* agar (Merck, Germany) supplemented with 7% fetal calf serum (Gipso, USA), 10% sheep blood (Jahad Daneshgahi, Tehran University, Tehran, Iran), polymyxin B, trimetoprim, amphotericin B, and vancomycin after homogenization.

### 2.4. Polymerase Chain Reaction (PCR)

22DNA extraction was performed by genomic DNA extraction kit (Bioneer, South Korea) followed by polymerase chain reaction (PCR) test with specific forward and reverse primers of* ureC* gene. Forward: 5′-CCCTCACGCCATCAGTCCCAAAAA-3′ and reverse: 5′-AAGAAGTCAAAAACGCCCCAAAAC-3′. Total volume of reactions was 25 *μ*L and solution included 2.5 *μ*L of 10x buffer (PH 8.4) containing 100 mM tris/HCl, 500 mM KCl, 2 mM MgCl_2_, 0.2 mM dNTP, 1.5 U Taq DNA polymerase, 2.5 *μ*L bacterial DNA, and 0.2 mM primer. Subsequently, 30 cycles performed at 94°C for 5 minutes (primary denaturation), 94°C for 1 minute, 55°C for 1 minute, and 72°C for 1 minute and final extension cycle performed at 72°C for 10 minutes.

### 2.5. Statistical Analysis

We used SPSS version 18.0 software for statistical analysis. *P* values less than 0.05 were regarded as statistically significant.

## 3. Results

Correlation between alarm symptoms such as epigastric pain when people are hungry with and without nocturnal awakening, bloating after meal with and without nocturnal awakening, and reflux with and without awakening with* H. pylori* detection from dental plaque and gastric biopsy has been shown in Table 1. Additionally, we observed recurrence of* H. pylori* infection among different samples which are listed in Table 1. All patients who received therapy already were again* H. pylori* positive while they were still carrying* H. pylori* in their dental plaque (*P* = 0.001). Correlation between endoscopic findings such as ulcerated and nonulcerated esophagitis, antral and erosive gastritis, gastric ulcer, duodenum ulcer, duodenitis, erosive duodenitis, and duodenum deformity with* H. pylori* isolation from dental plaques and gastric biopsies was shown in Table 2.

## 4. Discussion

*H. pylori* colonization affects more than half of the world's population and, thus, it is the most persistent bacterial infection worldwide. Various clinical manifestations are reporting about this persistence colonization [1]. Alarm symptoms of dyspepsia were found in 20–40% of community; thus, this disorder causes the annual millions of admission to the clinics [2]. Unfortunately, alarm symptoms have no good prognosis for* H. pylori* infection, but clinicians can apply this items to decide on who should receive early endoscopic service [11]. In current investigation, we compared the predictive value of alarm symptoms with detection of* H. pylori* in dental plaque and gastric biopsy. Actually, our research was the first which revealed that there is relation between epigastric pains and nocturnal awakening while patient is hungry. Interestingly, in 1989, for the first time, Krajden et al.isolated cultured* H. pylori* from the dental plaques, a finding which disclosed likely crucial role of oral cavity to feed the infectious load of stomach [12]. Undoubtedly, oral* H. pylori* is more difficult to be eradicated than gastric* H. pylori*, so a new adopted strategy to eradicate gastric* H. pylori* must be considered to target gastric* H. pylori* as well. Taken together, oral* H. pylori* seems to be the main source of gastric* H. pylori* for infection and reinfection models. Remarkably, all patients who received antibiotics were already* H. pylori* positive, while they were carrying* H. pylori* in their dental plaque (*P* < 0.05) (Table 1). To date, many studies had showed that* H. pylori* in dental plaque is a possible reservoir of recurrence of gastric infection and route of entrance of these bacteria to stomach from mouth [13]. Furthermore, some endoscopic findings such as ulcerated esophagitis, erosive gastritis, gastric ulcer, erosive duodenitis, and duodenum deformity had strong correlation with* H. pylori* isolation from gastric biopsies (*P* < 0.05) (Table 1). All of gastric biopsy's dyspeptic patients who had ulcerated esophagitis, erosive gastritis, gastric ulcer, and erosive duodenitis were* H. pylori* positive with cultivation and 75% of dyspeptic patients with duodenum deformity had this bacterium in gastric biopsies (Table 2). There are some investigations that compared between endoscopic findings and detection rate of* H. pylori.* In a study from the Republic of Georgia, it has been indicated that 78% and 58% of patients who had gastritis and peptic ulcer were* H. pylori* positive (*P* < 0.05) [14]. In an investigation in southern Iran, 70% and 86% of dyspeptic patients who had gastritis and duodenal ulcer were reported as* H. pylori* positive in their stomach, subsequently (*P* > 0.05). The exact mechanism of* H. pylori* reinfection in stomach is under debate but current novel findings showed that a prospective study which can check genomic content of both oral and gastric* H. pylori* can disclose more about unclear transmission route. Currently, there are various diagnostic tests to detect the infection with this mysterious microorganism, but there is no commonly acknowledged “gold standard” yet [1]. Similarly, our results showed that we might need to consider the current strategies to detect* H. pylori* in blood or biopsy samples. In other words, significant presence of* H. pylori* in oral cavity calls for urgent new assays to be tracked and likely possible eradicative intervention. However, we are still away from actual facts about this transmission. Thus, more research is needed to establish the exact transmission model of the infection of human population. Conclusively, our study showed that only successful eradication of gastric* H. pylori* cannot guarantee prevention of reinfection. Indeed, a new strategy which indicates concomitant eradication in oral and gastric colonization can cause prevention of* H. pylori* infection.

## Figures and Tables

**Table 1:** Relationship of clinical symptoms and frequency of  *H.  pylori* detected in dental plaque and gastric biopsy's dyspeptic patients.

Clinical symptoms	Dental plaque	PCR *ureC *	Gastric biopsy
	culture		culture
Epigastric pain without nocturnal awakening while patient is hungry (*n* = 82)	34 (41.4%)	62 (72.6%)	30 (36.6%)
Epigastric pain with nocturnal awakening while patient is hungry (*n* = 18)	15 (83.3%)	15 (83.3%)	16 (88.8%)
Bloating after meal without nocturnal awakening (*n* = 61)	39 (63.9%)	55 (90.1%)	34 (55.7%)
Bloating after meal with nocturnal awakening (*n* = 9)	5 (55.5%)	6 (66.6%)	6 (66.6%)
Reflux without nocturnal awakening (*n* = 31)	15 (48.4%)	24 (77.4%)	15 (48.4%)
Reflux with nocturnal awakening (*n* = 8)	6 (75.0%)	7 (87.5%)	6 (75.0%)
Previously infected with *H. pylori* and cured (*n* = 24)	18 (75.0%)	24 (100%)	13 (54.2%)

**Table 2:** Relationship of endoscopic findings and *H. pylori* prevalence in dental plaque and gastric biopsy's dyspeptic patients.

Endoscopic findings	Dental plaque	PCR *ureC *	Gastric biopsy
	culture		culture
Ulcerated esophagitis (*n* = 3)	1 (33.3%)	2 (66.6%)	3 (100.0%)
Nonulcerated esophagitis (*n* = 6)	6 (100.0%)	6 (100.0%)	0 (0.0%)
Antral gastritis (*n* = 21)	12 (57.1%)	17 (81.0%)	12 (57.1%)
Erosive gastritis (*n* = 25)	25 (100.0%)	25 (100.0%)	25 (100.0%)
Gastric ulcer (*n* = 8)	8 (100.0%)	8 (100.0%)	8 (100.0%)
Duodenitis (*n* = 24)	14 (58.3%)	20 (83.3%)	17 (70.8%)
Duodenum ulcer (*n* = 19)	15 (78.9%)	16 (84.2%)	12 (63.1%)
Erosive duodenitis (*n* = 12)	12 (100.0%)	12 (100.0%)	12 (100.0%)
Duodenum deformity (*n* = 16)	16 (100.0%)	16 (100.0%)	12 (75.0%)
